# QTL and Transcriptomic Analyses Implicate Cuticle Transcription Factor *SHINE* as a Source of Natural Variation for Epidermal Traits in Cucumber Fruit

**DOI:** 10.3389/fpls.2019.01536

**Published:** 2019-11-27

**Authors:** Stephanie Rett-Cadman, Marivi Colle, Ben Mansfeld, Cornelius S. Barry, Yuhui Wang, Yiqun Weng, Lei Gao, Zhangjun Fei, Rebecca Grumet

**Affiliations:** ^1^Department of Horticulture and Graduate Program in Plant Breeding, Genetics and Biotechnology, Michigan State University, East Lansing, MI, United States; ^2^Department of Horticulture, University of Wisconsin, Madison, WI, United States; ^3^USDA-ARS, Vegetable Crops Research Unit, Madison, WI, United States; ^4^Boyce Thompson Institute, Cornell University, Ithaca, NY, United States

**Keywords:** cucumber, Cucumis sativus, fruit surface, cuticle, lipid droplet, epidermis, fruit development

## Abstract

The fruit surface is a unique tissue with multiple roles influencing fruit development, post-harvest storage and quality, and consumer acceptability. Serving as the first line of protection against herbivores, pathogens, and abiotic stress, the surface can vary markedly among species, cultivars within species, and developmental stage. In this study we explore developmental changes and natural variation of cucumber (*Cucumis sativus* L.) fruit surface properties using two cucumber lines which vary greatly for these traits and for which draft genomes and a single nucleotide polymorphism (SNP) array are available: Chinese fresh market type, Chinese Long ‘9930’ (CL9930), and pickling type, ‘Gy14’. Thin-section samples were prepared from the mid-region of fruit harvested at 0, 4, 8, 12, 16, 20, 24 and 30 days post pollination (dpp), stained with Sudan IV and evaluated for cuticle thickness, depth of wax intercalation between epidermal cells, epidermal cell size and shape, and number and size of lipid droplets. ‘Gy14’ is characterized by columnar shaped epidermal cells, a 2–3 fold thicker cuticular layer than CL9930, increased cuticular intercalations between cells and a larger number and larger sized lipid droplets. In both lines maximal deposition of cuticle and increase in epidermal size coincided with exponential fruit growth and was largely completed by approximately 16 dpp. Phenotyping and quantitative trait locus mapping (QTL) of fruit sampled from an F_7_:F_8_ Gy14 × CL9930 recombinant inbred line (RIL) population identified QTL regions on chromosomes 1, 4 and 5. Strong QTL for epidermal cell height, cuticle thickness, intercalation depth, and diameter of lipid droplets co-localized on chromosome 1. SSR markers on chromosome 1 were used to screen for recombinants in an extended RIL population to refine the QTL region. Further fine mapping by KASP assay combined with gene expression profiling suggested a small number of candidate genes. Tissue specificity, developmental analysis of expression, allelic diversity and gene function implicate the regulatory factor *CsSHINE1/WIN1* as a source of natural variation for cucumber fruit epidermal traits.

## Introduction

Fruit surfaces play important roles in fruit development, maturation, and post-harvest quality. During growth and maturation, the exocarp is the first line of defense against herbivores, pathogens, and abiotic stresses such as dehydration, UV irradiation, and mechanical pressure. Following harvest, morphological features of the fruit surface can influence consumer preference and fruit quality. The epidermal cell structure can influence fruit firmness and susceptibility to damage. Cuticle structure and waxiness can modify external appearance such as glossiness and uniformity, and modulate rate of evaporative water loss, susceptibility to cracking and pathogen infection, and material penetration into the fruit surface (reviews: Hen-Avivi et al., 2014; Lara et al., 2014; Martin and Rose, 2014). These factors, in turn, influence handling practices in the commercial market chain.

For cucumber (*Cucumis sativus*), different market types vary substantially with respect to fruit surface features that influence consumer preferences, suitability for shipping, handling and storage, or performance demands for processing (pickling). Cucumber market types can vary considerably with regard to post-harvest longevity. Weight loss during the market chain is a primary concern and is influenced by epidermal properties including skin toughness and waxiness (Patel and Panigrahi, 2019; https://www.postharvest.net.au/product-guides/cucumber/). Wax load is inversely related with rate of water loss (Wang et al., 2015a), and untreated cucumber fruit can have a shelf life of less than a week (Patel and Panigrahi, 2019). As a result, packaging methods for fresh market cucumbers include wrapping in plastic, which is both expensive and environmentally undesirable, or treating the fruit with edible coatings.

The importance of the cuticle in product quality of fleshy fruits, coupled with relative ease of isolation from certain species, has driven studies focused on the biosynthesis and properties of fruit cuticles (Hen-Avivi et al., 2014; Lara et al., 2014; Martin and Rose, 2014). While much of the cuticle biosynthetic pathway has been established in Arabidopsis, characterization of mutants with altered cuticle composition (Isaacson et al., 2009; Nadakuduti et al., 2012; Yeats et al., 2012; Petit et al., 2014), tissue-specific transcriptomic analysis of developing tomato fruit peel (Mintz-Oron et al., 2008; Matas et al., 2011), and QTL mapping of introgression lines of wild tomato species (*Solanum pennelli*, *Solanum habroachaites*) (Cohen et al., 2017; Fernandez-Moreno et al., 2017) also have identified numerous genes associated with fruit cuticle development and composition. In cucumber, homologs of two key cuticle biosynthetic enzyme genes involved in cutin and wax biosynthesis, eceriferum (*CER1*) and *WAX2*, have been cloned (Wang et al., 2015b). Decreased expression of *CER1* and *WAX2* was associated with reduced wax load and increased water loss from harvested fruits.

Deposition of the cuticle and epicuticular waxes is developmentally programmed during organ growth to accommodate coverage required by increased surface area (Martin and Rose, 2014; Ingram and Nawrath, 2017). The precursors needed for cuticle and wax deposition are produced by epidermal cells and delivered to the fruit surface (Kunst and Samuels, 2003; Matas et al., 2010; Matas et al., 2011; Yeats and Rose, 2013; Huang 2018). Accordingly, *CsCER1* and *CsWAX2* are preferentially expressed in cucumber epidermal tissue (Ando et al., 2012; Wang et al., 2015a, b). For many species, cuticle deposition ceases during early fruit development, often before the fruit has reached maximum size and prior to the onset of ripening (Lara et al., 2014). We have observed that cuticle thickness in the pickling cucumber cultivar, ‘Vlaspik’, increases dramatically during the rapid growth phase from 4 to 16 days post-pollination (dpp) (Ando et al., 2012; Ando et al., 2015). The time period of 8–12 dpp also was marked by peak expression of genes associated with cuticle biosynthesis, such as several extracellular GDSL motif lipase/hydrolase proteins and lipid transfer proteins which have been implicated in lipid transport to extracellular surface (Ando et al., 2012).

Cuticle-related transcription factors have been identified from Arabidopsis, including the AP2 domain superfamily member, shine1 (SHN1), or win1 (WAX INDUCER1) (Aharoni et al., 2004; Broun et al., 2004). In tomato fruit, an exocarp-expressed *SHN* clade member, *SlSHN3*, regulates cuticle production; suppression of *SlSHN3* reduced cuticle production and caused a glossier fruit surface (Shi et al., 2013). In cucumber fruit, a preferentially peel-expressed homolog of *SHN1* (*CsaV31g030200*) exhibited peak transcription at 8–12 dpp, in concert with expression of the suite of cuticle biosynthesis associated genes (Ando et al., 2012). Several other transcription factors identified in tomato including MYB, MADS and homeodomain leucine zipper family members are also associated with regulation of production of cutin and wax components and cutin-localized secondary metabolites such as flavonols and terpenes (Adato et al., 2009; Isaacson et al., 2009; Gimenez et al., 2015). Many of the cuticle related transcription factors, including *AtSHN1/SlSHN3*, are also linked to epidermal cell patterning. In Arabidopsis and tomato *MIXTA-like* and leucine zipper transcription factors regulate both cuticle production and epidermal cell formation (Nadakuduti et al., 2012; Oshima et al., 2013; Lashbrooke et al., 2015a), and in maize, the glossy trait conferred by the AP2/EREBP transcription factor gene, *GL15,* influences epicuticular wax deposition, leaf hair formation, and cell shape (Moose and Sisco, 1996; Lauter et al., 2005).

While much of our understanding of epidermal cell structure and cuticle development has been derived from mutant or overexpression analyses using a limited number of model systems, little is known about the factors driving variation in natural populations (Petit et al., 2017). Current genetic resources and genomic tools can greatly facilitate our ability to identify and utilize sources of natural diversity across an increasing number of species, including cucumber. Reference genomes have been developed for representatives of two morphologically distinct cucumber market classes: the fresh market Chinese Long type, ‘CL9930’, and the American pickling type, ‘Gy14’ (Huang et al., 2009; Yang et al., 2012; Wang et al., 2018). In addition to obvious differences in fruit size and shape, CL9930 and Gy14 show markedly different epidermal and cuticle structures including amount and location of cuticle and wax deposition, number and size of lipid droplets present in epidermal cells, and size and shape of epidermal cells (Colle, 2015). In this study we sought to characterize epidermal cell growth and cuticle and wax deposition during cucumber fruit development, and identify genomic regions and candidate genes associated with variation using recombinant inbred lines (RILs) derived from progeny of Gy14 × CL9930. Several QTL were identified, including a major QTL on chromosome 1 associated with cuticle thickness, epidermal cell height, intercalation depth, and diameter of lipid droplets. Combined fine mapping, transcriptional analysis, and allelic diversity among cucumber accessions implicated the transcription factor *CsSHINE1/WIN1* as a regulator of natural variation for cucumber fruit epidermal traits.

## Materials and Methods

### Plant Materials and Growth Conditions

*Plant Materials*. Seed of cucumber (*C. sativus* L.) lines Gy14 (American pickling cucumber inbred line) and CL9930 (Chinese long type) were originally obtained from the University of Wisconsin and multiplied in the greenhouse. Pickling type cultivar Vlaspik was obtained from Seminis Vegetable Seed Inc, Oxnard, CA and American slicing type cultivar Poinsett 76 from Seedway, Hall NY. Our prior studies show that despite differences in size and shape, all four varieties exhibit a typical developmental pattern for cucumber with a period of cell division (∼0–4 dpp), followed by exponential growth, and approaching full size at 16–20 dpp (Ando et al., 2012; Colle, 2015; Colle et al., 2017). The three American varieties all have thick cuticles, while CL9930 has a thin cuticle (**Supplementary Table 1**)

*Developmental Study*. Gy14 and CL9930 plants were grown in the greenhouse (Michigan State University Plant Science Greenhouse Complex, East Lansing MI) in summer 2017 in 4 L plastic pots with Suremix Perlite soil medium (Michigan Grower Product, Inc., Galesburg, MI). The plants were watered and fertilized twice daily (with 44 ppm nitrogen of Peters Professional 20-20-20 General Purpose; Scotts, Marysville, OH) using an automated drip irrigation system (Dositron model D14MZ2, Clearwater FL). Supplemental high pressure sodium lights were used to provide a 16-h photoperiod. Pest and disease control were performed according to standard management practices in the greenhouse. When the plants initiated female flower production, a single ovary from each of 48 plants per line were hand-pollinated on the same day to ensure comparable environmental conditions for all fruit during development and provide sufficient fruit for each harvest date. Only one fruit was set per plant to have consistent developmental rates for all fruits by preventing competition for resources among fruits. At each sample date (0, 4, 8, 12, 16, 20, 24, and 30 dpp) three fruits per line (biological replicates)/age were harvested. Three samples (technical replicates) derived from the midsection of each fruit were prepared for microscopy.

*RIL Analyses*. A Gy14 × CL9930-derived F_7:8_ RIL population (Weng et al., 2015) that was previously genotyped by SNP array (Rubenstein et al., 2015) was grown in the greenhouse in Fall 2016, as described above. Each of 110 RILs and both parental lines were grown in triplicate (biological replicates) in a randomized, complete block design. Ovaries were hand pollinated and only one fruit per plant was set to minimize inter-fruit competition. Fruits were harvested at 16 dpp and prepared as described below. An extended F_7:8_ RIL population, consisting of 375 lines, was screened using SSR markers to identify recombinants for regions in chromosomes 1 and 4. Recombinant lines, parents, and reciprocal F_1_s were grown in triplicate (biological replicates) in the field in Summer 2018, in a randomized, complete block design at the Michigan State University Horticulture Teaching and Research Center, East Lansing, MI. Bee-pollinated flowers were tagged at anthesis. Fruit were harvested at 20–22 dpp and two samples (technical replicates) derived from the midsection of each fruit were prepared for microscopy as described below. Pest and disease control were performed according to standard management practices under field conditions. A subset of 17 RILs recombinant in the region of interest on chromosome 1 along with both parents were grown in the greenhouse in Spring 2019 under conditions described above to provide replication in different environments. Fruit were harvested at 16–20 dpp.

*RNA-Seq Experiment*. Fruit from the cultivars ‘Poinsett 76’ and ‘Gy 14’ were grown under greenhouse conditions as described above. Flowers were hand pollinated, such that 8 and 16 dpp fruit were harvested on the same day. Peels from three fruit (biological replicates) were harvested for each age and genotype and immediately frozen in liquid nitrogen and stored at −80°C until RNA extraction.

*CsSHN1 expression analysis*. Sixty plants of CL9930, Gy14, and Vlaspik were grown under greenhouse conditions as described above with the following modifications: supplemental lights provided an 18-h light cycle and plants were hand fertilized once a week. One or two flowers from the third to fifth node were hand-pollinated on the same day on each plant for each genotype; a single fruit was allowed to develop. Three fruits each from CL9930, Gy14 and Vlaspik were collected at anthesis, 4, 8, 12, 16, and 20 dpp.

### Microscopy and Measurement of Epidermal Traits

**Figure 1:**
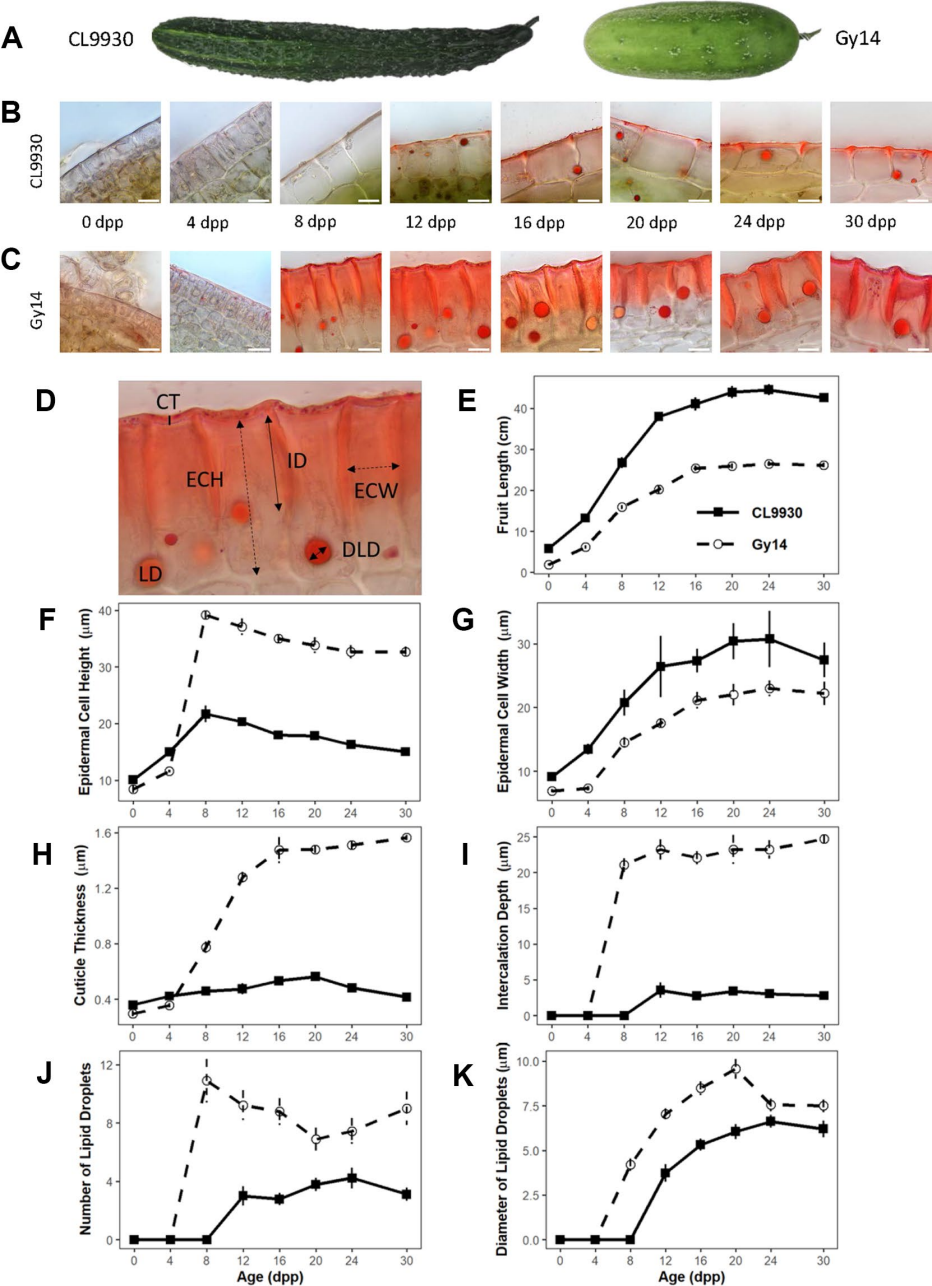
Developmental study of cucumber fruit epidermal traits. **(A)** CL9930 and Gy14 fruit at 16 days post pollination (dpp). **(B**, **C)** Cross sections of CL9930 **(B)** and Gy14 **(C)** at 0, 4, 8, 12, 16, 20, 24, and 30 days post pollination (dpp). Magnification = 1,000×. Scale bar = 10 µm. Samples were taken from the mid-section of the fruit. **(D)** Cross section illustrating traits measured; lipids were stained with Sudan IV. Cuticle thickness (CT) is represented by a solid, vertical line; Epidermal cell height (radial dimension, ECH) and Epidermal cell width (ECW) by dashed, double-headed arrows; Intercalation depth (ID) by a solid, double-headed arrow; LD indicates lipid droplet, and lipid droplet diameter (LDD) is represented by a solid, double-headed arrow. **(E)** Fruit length for CL9930 (solid line) and Gy14 (dotted line). **(F**–**K)** Developmental progression of fruit epidermal traits for CL9930 (solid line) and Gy14 (dotted line): **(F)** epidermal cell height (radial dimension), **(G)** epidermal cell width **(H)** cuticle thickness, **(I)** intercalation depth, **(J)** number of lipid droplets in 120 µm linear region of epidermal cells, **(K)** diameter of lipid droplets. Each value is the mean of 3 replicate fruits ± S.E.

A wedge (∼1 cm^3^) was cut from the mid-section of each fruit and sliced to ∼0.1 mm thickness by a sliding block microtome. All methods pertaining to staining with Sudan IV (Sigma-Aldrich, St. Louis MO) and subsequent washing were performed according to the methods Buda et al. (2009) with the exception of RIL experiments (Summer 2018 and Spring 2019) when samples were mounted in glycerin (Columbus Chemical Industries, Columbus WI) instead of distilled water. All samples in water were imaged by microscopy the same day; glycerin mounted samples were imaged within one week. Images for the RIL population from the Fall 2016 experiment were captured using an EVOS FL Auto imaging system (ThermoFisher Scientific; http://www.thermofisher.com) with 400× magnification and analyzed using the Nikon NIS-Elements BR imaging system. For the developmental study (Summer 2017) and the extended RIL population (Summer 2018, Spring 2019), images were obtained using a Nikon Eclipse Ni-U microscope and Nikon DS-Fi3 camera (Nikon Instruments Inc.; Melville, NY) at 600× and 200× magnification, respectively. Epidermal features were measured as shown in **Figure 1**. To allow for better comparison among samples and avoid influence of warts and spines, all measurements were made in areas between spines. To standardize measurements of epidermal features, a line of 120 µm (developmental study) or 450 µm (RIL populations) was drawn across a given sample and features were measured within that area. Three measurements across the sample were taken for cuticle thickness (CT), intercalation depth (ID), and epidermal cell height (ECH); the mean value was used in subsequent analyses. Epidermal cell width (ECW) was determined by dividing 450 µm by the number of epidermal cells in that area. The number of lipid droplets (NLD) were counted in the given area. The diameters of all lipid droplets (DLD) in this area were measured and the average used in subsequent analyses. Calculation of Pearson’s correlation coefficients among traits were conducted using the R package ‘GGally’ (https://github.com/ggobi/ggally).

### Mapping of Epidermal Traits

#### QTL Analyses

For QTL analysis, a subset of 916 unique markers were used from a previously constructed genetic map (Rubenstein et al., 2015; Weng et al., 2015). Composite interval mapping (CIM) was performed with QTL Cartographer v2.5 using the standard model Zmapqtl 6 with walking speed of 1 cM, 5 background markers, and window size of 5 cm (Wang et al., 2012). The forward and backward method was used to select markers as cofactors. The LOD significance threshold was determined by a 1,000-permutation test at 5% probability.

#### SSR Screening

Microsatellite (SSR) markers within the 2.0-LOD intervals at the two QTL loci on Chr1 and Chr4 were used to genotype an expanded population of 375 F_7:8_ Gy14× 9930 RILs to identify recombinants between the flanking markers. Due to the inconsistency in physical locations of the flanking SNP markers in 9930 v2.0 and Gy14 v2.0 draft genome assemblies, multiple SSR markers in the two target regions were employed. Information of markers used to identify recombinants is provided in **Supplementary Table 2**. DNA extraction, PCR amplification of molecular markers and gel electrophoreses followed Gao et al. (2016).

#### KASP™ Screening

*DNA Isolation and Quantification.* Tissue samples (∼50 mg) from young leaf tissue of cucumber seedlings (∼1–2 weeks) were lyophilized in a freeze-dryer and ground into fine powder with a high-throughput homogenizer (OPS Diagnostics, Lebanon, NJ). DNA was isolated and quantified as described in Wiersma et al., 2017. Briefly, DNA was isolated using the Mag-Bind^®^ Plant DNA Plus 96 Kit (M1128, Omega Bio-Tek,Norcross, GA) on a King Fisher Flex Purification System (Thermo Scientific, Waltham, MA). DNA was quantified using the Quant-iT^™^PicoGreen^®^ dsDNA Kit (Life Technologies Corp., Grand Island, NY) on a CFX384 Real-Time thermal cycler, C1000 (BioRad, Hercules, CA).

*SNP Calling and KASP* ^™^ *Assay*. From the expanded RIL population, 87 lines selected to be recombinants in QTL regions 1 or 4 were grown in triplicate in field conditions and phenotyped as described above. Due to the strength of the QTL on chromosome 1, further fine mapping was performed using the 29 lines that were identified by SSR assay to be recombinants in that region. The draft genomes of Gy14 (Version 2, cucurbitgenomics.org) and CL9930 (Version 3, cucurbitgenomics.org) were aligned using the *nucmer* function of MUMmer 4 (Marçais et al., 2018). Single nucleotide polymorphisms (SNPs) were then called using the *show-snps* function. SNPs immediately flanking the region of interest on chromosome 1 and at an interval of 0.25 Mb along this region were used to design allele specific forward KASP^™^ (LGC, Teddington, Middlesex, UK) and common reverse primers, where all CL9930 alleles would express a FAM signal, while the Gy14 allele would express a HEX signal. KASP^™^ markers that met the following criteria were selected for use in assay: GC content of 30–55%; approximate melting temperature of 64 ± 2°C; length of 21–28 bp; product size of 50–100 bp; limited to no secondary structure or repeats; and GC clamp with no more than 3 Gs or Cs in the last 5 bp of the primer (**Supplementary Table 3**). PCR thermocycling and fluorescence detection was conducted using a CFX384 Real-Time thermal cycler (BioRad), where alleles were determined using the CFX manager software (v.3.1).

### Expression Analyses

#### RNA-Seq Experiment

*Sample Collection and RNA Extraction*. Peels were collected from 8 and 16 dpp of ‘Poinsett 76’ and ‘Gy 14’ using a vegetable peeler, immediately frozen in liquid nitrogen and stored at −80°C until further use. Three fruit (biological replicates) were collected for each age and genotype. Peel samples were ground in liquid nitrogen using a mortar and pestle. RNA extraction was performed using the MagMAX Plant RNA Isolation Kit protocol (Thermo Fisher Scientific, Waltham MA) with the exception of increased amount of tissue and buffer; approximately 100–150 mg tissue was transferred to a 1.5 ml tube with 1,000 µl of lysis buffer. After lysis and centrifugation as per the protocol, supernatant was transferred to a 96-deep-well plate for high-throughput RNA extraction, on a KingFisher Flex Purification System. Immediately after the run was complete, the 96-well plate was transferred to storage at −80°C. RNA concentration and quality were measured using Qubit 2.0 Fluorometer (Thermo Fisher Scientific) and LabChip GX (Perkin Elmer, Waltha MA), respectively. All samples had a minimum RNA quality score of 8.

*RNA-Seq Library Preparation and Sequencing.* RNA-seq libraries were prepared at Michigan State University’s Research Technology Support Facility, using the Illumina TruSeq Stranded mRNA Library Preparation Kit on a Sciclone G3 workstation following manufacturer’s recommendations. An additional cleanup with 0.8× AmpureXP magnetic beads was performed after completion of library preparation. Quality control and quantification of completed libraries was performed using a combination of Qubit dsDNA HS and Advanced Analytical Fragment Analyzer High Sensitivity DNA assays. The libraries were divided into two pools of 15 libraries each. Pools were quantified using the Kapa Biosystems Illumina Library Quantification qPCR kit. Each pool was loaded onto one lane of an Illumina HiSeq 4000 flow cell and sequencing was performed in a 1 × 50 bp single read format using HiSeq 4000 SBS reagents. Base calling was done by Illumina Real Time Analysis (RTA) v2.7.7 and output of RTA was demultiplexed and converted to FastQ format with Illumina Bcl2fastq v2.19.1.

*Differential Expression Analysis.* Reads were cleaned, and adaptor sequences were removed using Trimmomatic v. 0.34 (Bolger et al., 2014) with the following settings: LEADING:3 TRAILING:3 SLIDINGWINDOW:4:15 MINLEN:35. Quality control was performed using FastQC (http://www.bioinformatics.bbsrc.ac.uk/projects/fastqc). A cucumber transcriptome fasta file was made from the ‘Chinese Long’ (v2) (Huang et al., 2009; Li et al., 2011) genome using the *gffread* function from the cufflinks software package (Trapnell et al., 2010) and high-quality reads were then quasi-mapped to the transcriptome using Salmon v. 0.9.1 (Patro et al., 2017) with default settings.

Read quantification data was imported into R using the tximport R package (Soneson et al., 2015) and differential expression analysis was performed using DEseq2 (Love et al., 2014) with log-fold-change-shrinkage. Age and genotype were combined into a single factor for differential expression analysis and contrasts between the four conditions (‘Poinsett 76’ 8 dpp, ‘Poinsett 76’ 16 dpp, ‘Gy14’ 8 dpp, ‘Gy14’ 16 dpp) were performed. Differentially expressed genes were called significant using an adjusted p-value (Benjamini and Hochberg, 1995) of less than 0.05. A cutoff expression change of above two-fold was used to define biological significance. Expression data for candidate genes from CL9930 was accessed using the gene expression profiles function (Zheng et al., 2019) of http://cucurbitgenomics.org/; gene expression project PRJNA 312872 (Wei et al., 2016).

#### Expression Analysis of *CsSHN1* During Fruit Development

Pericarp samples isolated from the middle part of the fruit of CL9930, Gy14 and Vlaspik were immediately frozen in liquid nitrogen. Total RNA samples from the pericarp tissue were prepared using the Trizol method (Thermo Fisher Scientific), followed by DNase I treatment and clean up (Qiagen, Germantown MD). The amount of RNA for each sample was measured using the nanodrop ND-1000 (Thermo Fisher Scientific). First strand cDNA synthesis was performed using the High Capacity RNA-to-cDNA Kit (Thermo Fisher Scientific) and by following the protocol described by Ando and Grumet (2010). Gene-specific primers were designed using Primer Express software (Applied Biosystems, Forest City CA). The ABI Prism 7900HT Sequence Detection System was used for qRT-PCR analysis. Revolution PCR Master Mix (Integrated Scientific Solutions, San Diego CA) with ROX as reference dye was used for gene amplification. *C. sativus polyubiquitin* (CuSa200910_13711) was used as an endogenous control for normalization. Expression of target genes was assessed with reference to corresponding standard curves. qRT-PCR was performed using cDNA of three fruits (three biological replicates)/genotype with three technical replicates/biological replicate. Data were analyzed by analysis of variance (ANOVA) and Tukey HSD protocol in SAS (SAS Institute, Cary, NC).

#### Analysis of *CsSHN1* Alleles

*Identification of CsSHN1 Alleles in Gy14, Poinsett 76 and Vlaspik.* DNA was extracted from three lines ‘Gy 14’, ‘Poinsett 76’, and ‘Vlaspik’ using the Kingfisher DNA extraction robot as described in Wang et al. (2018). After quantitation, all libraries were pooled in equimolar amounts which was loaded on one lane of an Illumina HiSeq 2500 High Output flow cell (v2) alongside other samples with a targeted coverage of ∼30×. Sequencing was carried out using HiSeq SBS reagents in a 2 × 150 bp paired end format (PE150). Reads were cleaned and adaptor sequences were removed using Trimmomatic v. 0.33 (Bolger et al., 2014). Reads were mapped to the ‘Chinese Long 9930’ (v2) (Huang et al., 2009; Li et al., 2011) cucumber genome using BWA-MEM (Li and Durbin, 2009). Duplicate reads were marked with Picard (https://broadinstitute.github.io/picard/) and the GATK “Best Practices” pipeline was used for variant calling (McKenna et al., 2010; DePristo et al., 2011; Van der Auwera et al., 2013). Variants were hard-filtered with the GATK base recommendations. Initial analyses were done with CL9930v2 but nucleotide positions were later converted to CL9930v3.

*Survey of Cucumber Germplasm for CsSHN1 Alleles.* Cucumber accessions for which resequencing data were available were examined for the nucleotide present at position 16961026 within the *CsSHN1* locus (*CsaV3_1G030200*). Sequence data for 115 accessions were available from Qi et al. (2013). Data for an additional 89 accessions (**Supplementary Table 4**), comprising a portion of the cucumber core outlined in Wang et al., 2018, were also analyzed. Samples were included for which there were at least 10 reads at position 16961026.

## Results

### Developmental Progression of Fruit Epidermal Traits

The parental inbred lines Gy14 (a pickling breeding line) and CL9930 (an Asian fresh market breeding line) differ for fruit size, shape, and epidermal properties (**Figure 1**). Epidermal cells of Gy14 have a palisade orientation, thicker cuticle, and deeper cuticular intercalations between cells, whereas CL9930 has a flatter epidermal cell shape, with wider cells, thinner cuticle and minimal cuticular intercalation. An additional striking feature of the epidermal cells was the presence of large circular droplets brightly stained with the red lipid-soluble dye, Sudan IV. The number and size of the lipid droplets also differed between the two lines of interest, with larger and more numerous lipid droplets in Gy14.

A developmental study was performed in the greenhouse to assess changes in epidermal properties during fruit growth and maturation. To minimize effects of competition on growth rate, a single fruit was set per plant. Fruit were harvested at 0 (anthesis), 4, 8, 12, 16, 20, 24, and 30 dpp (maturity). Subsequent to initial fruit set and the period of active cell division (0–4 dpp) (Fu et al., 2010; Ando et al., 2012; Colle et al., 2017), fruit epidermal properties changed dramatically, especially in Gy14 (**Figures 1F–K**). Increases in epidermal cell height and width, cuticle thickness and intercalation between epidermal cells, and lipid droplet number and size, generally showed a sigmoidal trend with fruit age. The greatest increases for most traits occurred between 4 and 12 dpp, coinciding with the period of exponential fruit growth (**Figure 1E**). Differences between Gy14 and CL9930 became apparent for most traits between 4 and 8 dpp and were largely stabilized by 16 dpp. Obvious intercalations and appearance of lipid droplet were observed sooner in Gy14, at 8 dpp, rather than 12 dpp in CL9930.

### Fruit Epidermal QTLs

**Figure 2:**
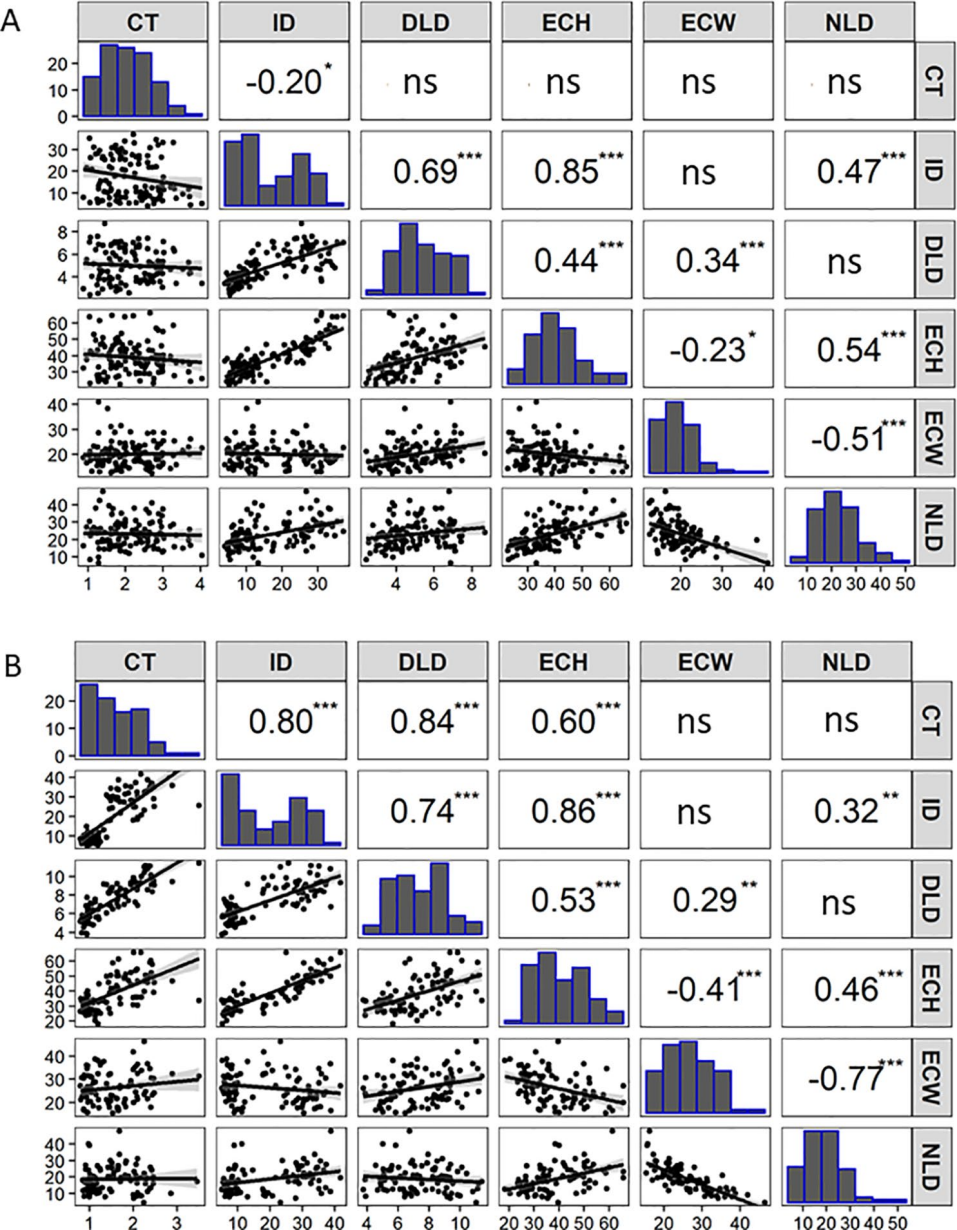
Correlation matrix containing scatterplots, distributions, and Pearson’s correlation coefficients of the cucumber fruit epidermal traits for: **(A)** RIL population (F_7:8_) of Gy14 × CL9930 (n = 110 lines, 16 dpp) grown in the greenhouse in Fall 2016. **(B)** Recombinant RIL lines (n = 87 lines, 20 dpp) grown in the field in 2018. Cuticle thickness (CT), Intercalation depth (ID), Diameter of lipid droplets (DLD), Epidermal cell height (ECH), Epidermal cell width (ECW), and Number of lipid droplets (NLD) in 450 µm linear region of epidermal cells. (*ns* not significant; *P < 0.05; **P < 0.01; ***P < 0.001. (Each value is the mean of three replicate fruit per RIL).

An F_7:8_ Gy14 × CL9930 RIL population consisting of 110 lines was grown in the greenhouse in 2016 and evaluated for cucumber fruit epidermal traits as described above. Based on the observations of the developmental study, fruit were harvested at 16 dpp, after growth had stabilized and differences in fruit epidermal were readily observable. Phenotypic distributions and correlations among the traits are summarized in **Figure 2A**. Strong, positive correlations were observed among intercalation depth, epidermal cell height, and diameter and number of lipid droplets. Epidermal cell width was negatively correlated with epidermal cell height and number of lipid droplets.

**Figure 3:**
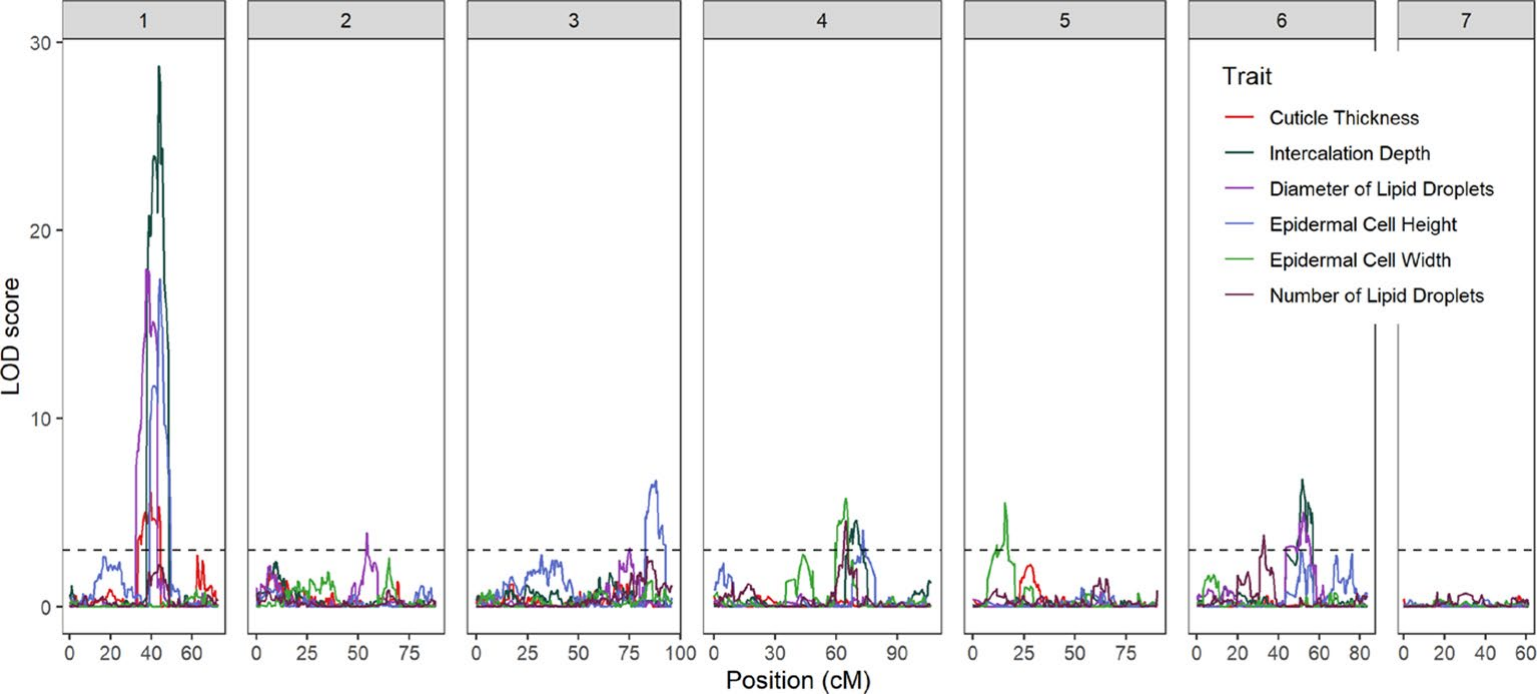
LOD profiles of fruit epidermal QTLs detected in the RIL population across the seven chromosomes of cucumber. The dashed horizontal line is LOD threshold = 3.0.

Fourteen QTL were detected on six of the seven cucumber chromosomes (**Figure 3** and **Table 1**). On chromosome 1, a major QTL, *ECT1.1* (epidermal cell traits) was detected for cuticle thickness, epidermal cell height, intercalation depth, and diameter of lipid droplets that explained 18.4%, 38.1%, 44.1%, and 37.9% of the phenotypic variation for each trait, respectively. A single QTL was found on chromosome 2 for diameter of lipid droplets; chromosome 3 contained one QTL for epidermal cell height; and chromosome 4 had a single QTL for epidermal cell height, intercalation depth, epidermal cell width, and number of lipid droplets. In addition to the QTL for epidermal cell width found on chromosome 4, there also was a QTL detected on chromosome 5. Lastly, several QTL were found on chromosome 6 for intercalation depth, diameter of lipid droplets, and number of lipid droplets. In each case where there were multiple QTL for a single trait, the percent variation explained was greatest for the QTL on chromosome 1.

**Table 1:** Summary of fruit epidermal QTLs detected: Cuticle thickness (CT), Epidermal cell height (ECH), Intercalation depth (ID), Diameter of lipid droplets (DLD), Epidermal cell width (ECW), and Number of lipid droplets (NLD).

**Trait**	**Chr**	**QTL Loci**	**Peak Pos (cM)**	**Nearest Marker**	**LOD Score**	**2.0 LOD Interval**				**Additive Effect (a)**	**Percent Variation (%) R** **^2^**
						**Left Locus**	**Left cM**	**Right Locus**	**Right cM**		
CT	1	*qCT1.1*	39.8	SNP.120657	6.1	SNP.10249	37.9	SNP.9737	43.1	0.34	18.4
ECH	1	*qECH1.1*	44.1	SNP.9649	17.4	SNP.9649	43.9	SNP.9341	44.9	−6.40	38.1
	3	*qECH3.1*	87.8	SNP.31473	6.7	SNP.32685	82.8	SNP.117081	89.1	3.60	11.5
	4	*qECH4.1*	73.0	SNP.128649	4.1	SNP.55545	71.6	SNP.146469	77.3	−2.69	6.7
ID	1	*qID1.1*	43.6	SNP.9649	28.7	SNP.9649	43.5	SNP.9649	44.1	−7.50	58.3
	4	*qID4.1*	69.8	SNP.55081	4.6	SNP.140945	67.9	SNP.128649	72.6	−2.07	4.5
	6	*qID6.1*	51.8	SNP.85409	6.8	SNP.84997	50.7	SNP.86689	53.1	−2.66	6.9
DLD	1	*qDLB1.1*	37.4	SNP.9941	18.0	SNP.9941	37.0	SNP.10249	37.9	−0.94	42.5
	2	*qDLB2.1*	54.2	SNP.143745	3.9	SNP.23461	52.8	SNP.131577	56.5	0.38	6.9
	6	*qDLB6.1*	52.2	SNP.175561	5.0	SNP.84997	50.8	SNP.128265	53.6	−0.44	8.8
ECW	4	*qECW4.1*	64.6	SNP.52733	5.8	SNP.55017	61.8	SNP.53205	65.7	2.23	14.8
	5	*qECW5.1*	15.7	SNP.76545	5.5	SNP.134473	15.2	SNP.77021	17.0	2.09	14.5
NLD	4	*qNLB4.1*	64.6	SNP.52733	4.6	SNP.122297	63.9	SNP.53205	65.5	−2.96	12.6
	6	*qNLB6.1*	32.8	SNP.151005	3.8	SNP.90761	30.8	SNP.89757	35.6	−3.53	10.3
*Negative additive effect values (a) indicate that the allele is derived from parent Gy14. Positive additive effect values (a) indicate that the allele is derived from parent CL9930.*											

### Marker-Assisted Screening and Fine Mapping of Chromosome 1

**Figure 4:**
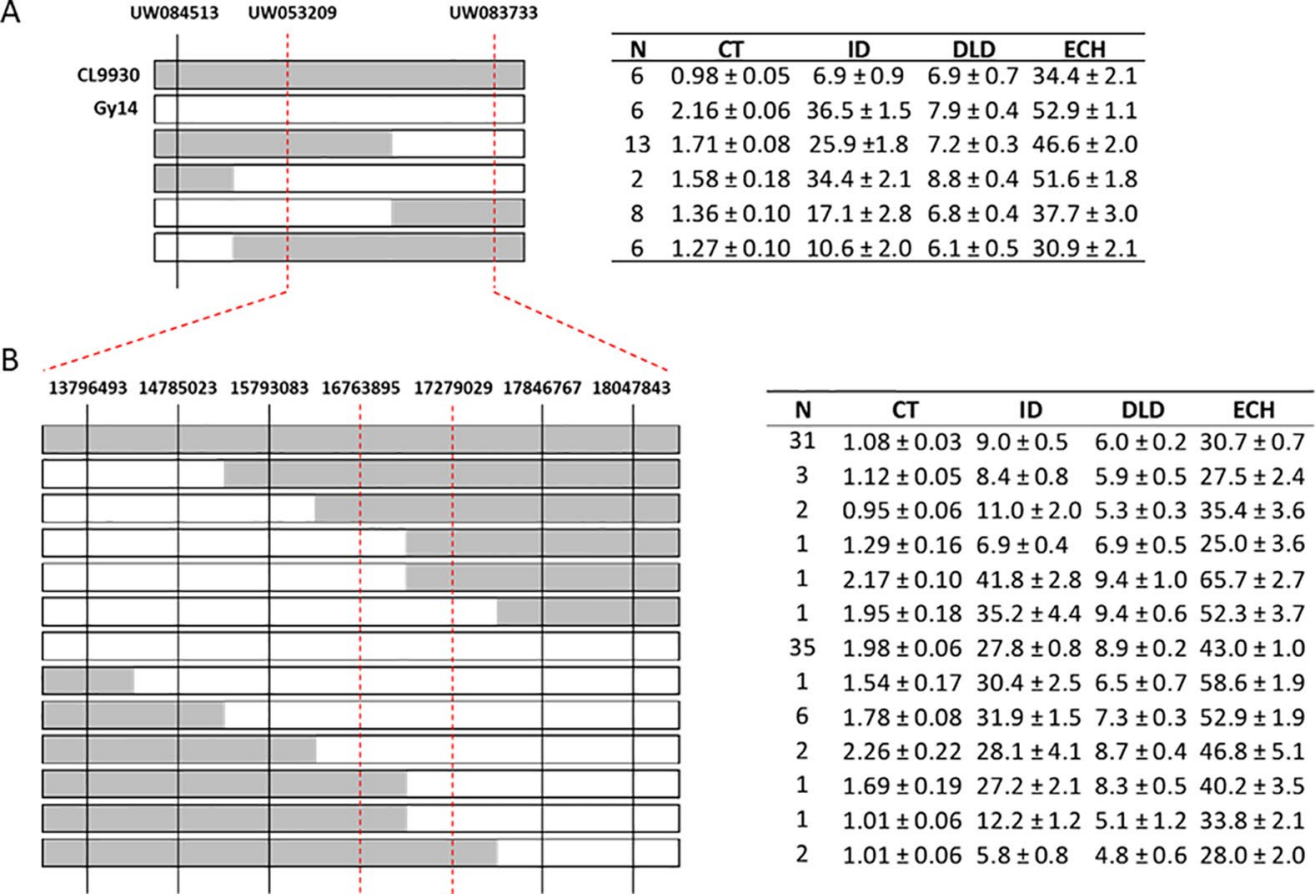
Fine mapping of chromosome 1. **(A)** Genotyping of recombinant plants from an expanded F_7:8_ RIL population using SSR markers (n = 375); 87 lines were selected of which 29 were recombinant in the region of interest on chromosome 1. Parental lines, CL9930 and Gy14, are included as reference. CL9930 alleles are denoted by grey, Gy14 by white. **(B)** Additional genotyping of expanded RIL population using KASP™ markers in narrowed region of chromosome 1. Phenotypes of RILs sharing a given recombinant type are indicated in the adjacent tables. Measurements of traits are as for **Figure 3**. Trait values (µm) are the mean ± S.E. of all RILs sharing a given recombinant type (three biological replicates/RIL).

The strongest QTL were detected on chromosome 1, with LOD scores in the range of 6.1–28.7. Linkage analysis also supported the QTL on chromosome 1 (**Supplementary Table 5**). To narrow the region of interest, SSR markers were designed to flank the peak (at positions 14516668 and 18050191 CL9930 genome v3) with an additional marker in between (position 14783187). These markers were then used to screen an expanded F_7_:_8_ RIL population (n = 375) to identify recombinant individuals in the region of interest. Of these, 87 lines were selected, including 29 identified as recombinant in the designated region on chromosome 1 (**Figure 4A**).

Selected lines, parents and reciprocal F_1_s (Gy14 × CL9930 and CL9930 × Gy14) were grown in the field in 2018 and phenotyped at 20–22 dpp. With the exception of diameter of lipid droplets, the both F_1_’s showed intermediate phenotypes relative to the parents (**Supplementary Figure 1**). Similar patterns of distribution and correlations among traits were observed for RILs as for the 2016 experiment in the greenhouse, with the exception of cuticle thickness, likely due to better imaging equipment in 2018 that allowed for more accurate determination of cuticle thickness (**Figure 2B**). In the 2018 experiment, cuticle thickness was strongly and positively correlated with intercalation depth, diameter of lipid droplets, and epidermal cell height (**Figure 2B**). The observed correlations among these four traits were consistent with their overlapping QTL positions on chromosome 1.

Combining phenotype data for the four traits with the SSR genotypic data, the region of interest on chromosome 1 was narrowed to approximately 3 Mb (14.78 Mb to 18.05). Recombinant individuals were then genotyped within this region with a set of seven SNP-based KASP^™^ markers spaced at approximately 0.5 Mb intervals. This narrowed the region of interest to 512 kb, from 16.76 to 17.28 Mb (**Figure 4B**). A subset of RILs recombinant in this region of chromosome 1 was also grown in the greenhouse in Spring 2019 to test expression of phenotype in different environments. Analysis of data from field Summer 2018 and greenhouse Spring 2019 showed very highly significant correlations (all P values <2.0E−06) between the two conditions for all four traits (cuticle thickness—r = 0.99; intercalation depth r = 0.96; diameter of lipid droplets r = 0.84; epidermal cell height r = 0.86) (**Supplementary Table 6**).

### Candidate Genes Influencing Cucumber Fruit Epidermal Properties

The 0.51 Mb KASP marker-defined region on chromosome 1 contained 25 annotated genes (CL v3; http://cucurbitgenomics.org/). To refine the list of candidates we performed RNA-seq on fruit peels of the parental lines and further utilized existing expression data from our prior work comparing peels from 8 dpp and 16 dpp Gy14 fruit (Mansfeld et al., 2017) and expression data from Wei et al. (2016) comparing a wide variety of cucumber tissue types in CL9930 (accessed *via* http://cucurbitgenomics.org/, Gene expression project PRJNA 312872). As we were dealing with fruit epidermal related traits, and based on the developmental analyses showing that increase in cuticle related traits occurred most rapidly between 4 and 12 dpp, two criteria were used to filter the genes: (i) preferentially expressed in peel vs. flesh; and (ii) elevated expression at 8 dpp relative to 16 dpp. Of the 20 genes in this region showing expression in fruit, four had greater expression in peels than in flesh: *CsaV31g030090*, a putative heme oxygenase, associated with chlorophyll degradation; *CsaV31g030200*, a homolog of the cuticle-related transcription factor, *SHN1/WIN1*; *CsaV31g030210*, a gene with unknown function; and *CsaV31g030360,* a glucan endo-1,3-beta glucosidase (**Table 2**). These genes also showed greater expression in exocarp vs. mesocarp in our prior studies of pickling cucumber cv. Vlaspik, which also has a thick cuticle (Ando et al., 2015). Of the four genes, specificity to the peel was much stronger for *CsSHN1*, approximately 80-fold vs. 2-fold for *CsaV31g030090*, *CsaV31g030210*, and *CsaV31g030360*. Furthermore, *CsSHN1* was essentially exclusively expressed in fruit peel relative to other tissues and organs. In contrast, *CsaV31g030090* exhibited approximately 25-fold higher expression in flowers than fruit; *CsaV31g030210* exhibited 50–100-fold higher expression in roots, leaves, and flowers than in fruit; and *CsaV31g030360* was expressed comparably throughout the plant.

**Table 2:** Annotated genes within fine-mapped region of QTL on cucumber chromosome 1 (16.764–17.279 Mb). Chromosomal positions and annotations are derived from Chinese Long v. 3.

Gene ID	Fruit expression (2 wks post-pollination)		Peel expression		Description	Arabidopsis Homolog	
	CL9930^a^		Gy14^b^				
	Flesh	Peel	8 dpp	16 dpp			e Value
*CsaV31g030010*	16	12	17.5	91.5	nitrate transporter	At2g26690	2.5 e−245
*CsaV31g030020*	12.2	9.7	11.2	15.1	similarity to DNA helicase	At1g65810	7.3 e−23
*CsaV31g030030*	0	0	0	0	Zn binding dehydrogenase family	At5g16990	3.5 e−23
***CsaV31g030090***	***13.9***	***21.7***	57.7	42.9	heme oxygenase	At2g26670	9.2 e−111
*CsaV31g030110*	8.5	2.2	2.4	4.7	metal transporter	At5g53550	3.2 e−286
*CsaV31g030120*	0	0	2.6	0.9	peroxidase superfamily	At2g41480	8.4 e−95
*CsaV31g030140*	0	0	0	0	TIR-NBS-LRR protein	At5g17680	7.2 e−157
*CsaV31g030160*	14.1	19.8	0	0	unknown	no match	
*CsaV31g030170*	0	0	0	0	peroxidase superfamily	At2g41480	4.3 e−117
*CsaV31g030190*	7.8	9.2	9.1	5.5	SPX domain containing	At2g26660	1.0 e−100
***CsaV31g030200***	***1.4***	***115.8***	***343.5***	***13.3***	SHINE1/WIN1	At1g15360	1.2 e−57
***CsaV31g030210***	***8.3***	***18.5***	7.2	11	unknown	At1g52565	2.1 e−12
*CsaV31g030220*	124.2	99.5	86.7	99.8	50S ribosomal L36	At5g20180	2.3e−30
*CsaV31g030230*	28.4	21.7	40.8	30.7	DUF 4050 family protein	At5g25360	2.5e−58
*CsaV31g030250*	0.1	0.5	0.5	0.1	EIN3 binding F-box protein-like	At5g25350	1.8e−84
*CsaV31g030300*	175.0	128.5	100.7	106.2	tetratricopeptide repeat protein	At5g20190	2.1e−25
*CsaV31g030310*	0.9	0.7	0.3	6.4	nucleotide transporter like	At1g02630	1.0e−133
*CsaV31g030330*	22.9	28.2	14.5	23.7	nuclear pore complex protein	At5g20200	7.8e−82
*CsaV31g030340*	0	0	0	0	axial regulator YABBY-like	At2g26580	2.4e−62
*CsaV31g030350*	8.7	8.1	13.8	7.6	methyl CpG binding domain	At3g15790	3.4e−29
***CsaV31g030360***	***6.8***	***13.1***	15.5	16.3	glucan endo-1-3 beta glucosidase	At2g26600	3.4e−29
*CsaV31g030390*	0.2	0	2.1	11.1	blue copper protein	At5g20230	4.7 e−16
*CsaV31g030400*	11.4	11.3	12.9	14.9	transducin family protein	At2g26610	0
*CsaV31g030420*	12.4	13.9	14.6	12.4	acetylgluosaminyl transferase family	At2g13290	6.8 e−143
*CsaV31g030440*	0	0	0	0	pentatricopeptide repeat containing	At4g02750	1.2 e−15

Predicted genes with no homology match and no expression in fruit were not included.

a Expression data from Wei et al., 2016. Accessed via http://cucurbitgenomics.org/, Gene expression project PRJNA 312872.

b Expression data from Mansfeld et al., 2017. Available via http://cucurbitgenomics.org/, Gene expression project PRJNA 345040.

Bold indicates higher expression in peel than flesh and higher expression at 8 dpp than 16 dpp.

**Figure 5:**
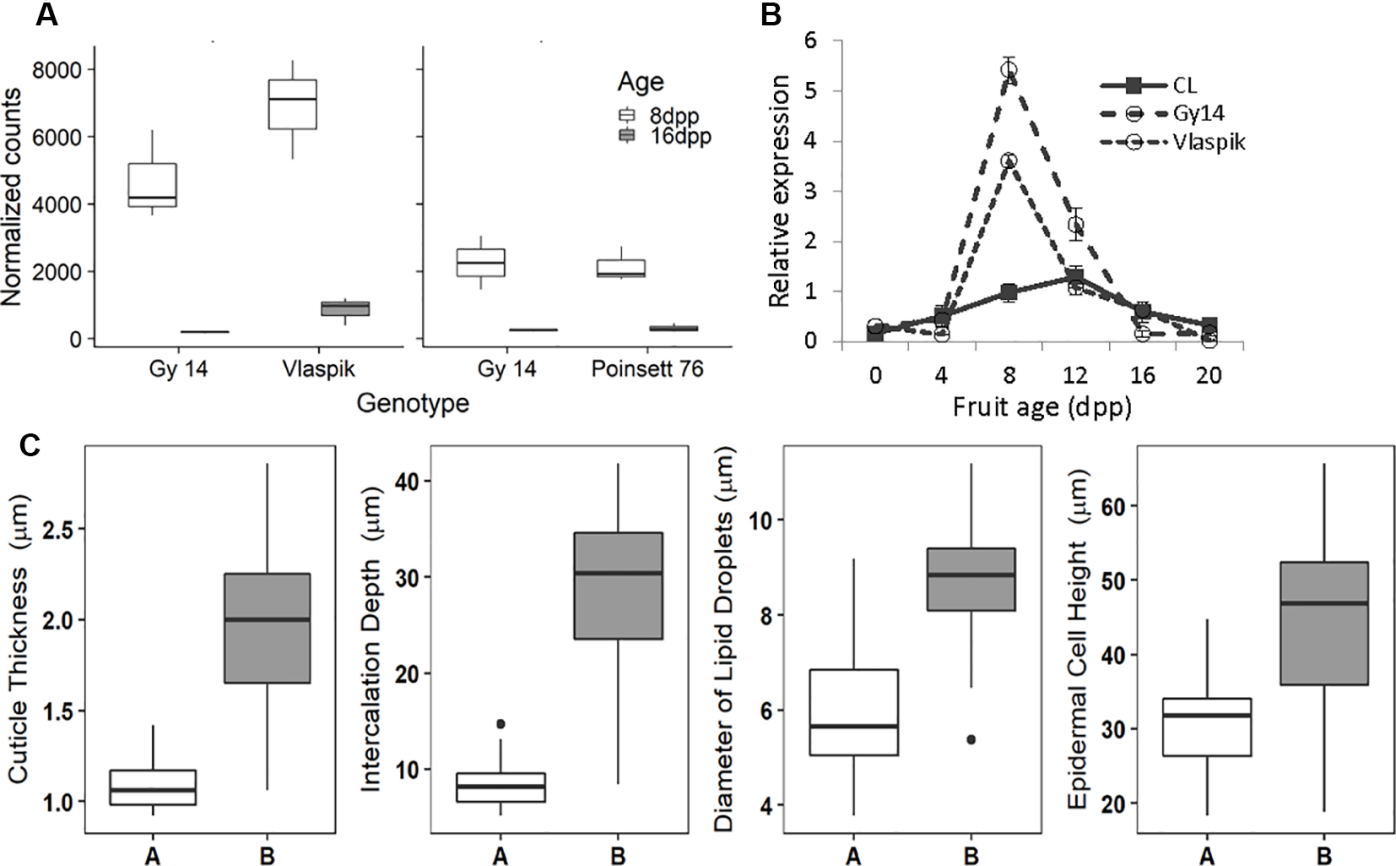
Transcriptional analysis and allele effect of the CsSHINE1 (CsaV3_1g030200). **(A)** Comparison of expression of *CsSHN1* in fruit peel from cucumber fruit at 8 and 16 days post pollination (dpp). **(B)** Expression of *CsSHN1* in CL9930 and two pickling cultivars, Gy14 and Vlaspik, during cucumber fruit development, from anthesis (0 dpp) to 20 dpp. Each value is the mean of 3 biological replicates with 3 technical replicates/biological replicate ± S.E. **(C)** Allele effect of *CsSHN1* SNP at position 16961026 (in Chinese Long V3) for cuticle thickness, intercalation depth, diameter of lipid droplets and epidermal cell height as assessed from the expanded RIL population. ‘A’ refers to RILs with CL9930 allele (n = 25) and ‘B’ to RILs with the Gy14 allele (n = 35).

With regard to fruit development, *CsSHN1* was the only gene in the QTL1 region with significantly higher expression in peels of 8 dpp fruit than 16 dpp fruit (**Table 2**). Higher expression (P < 0.05 and 2-fold difference) of *CsSHN1* at 8 dpp than 16 dpp also was observed in the cultivars ‘Vlaspik’ and ‘Poinsett 76’ (**Figure 5A**). Examination of *CsSHN1* expression during cucumber fruit growth from 0–20 dpp showed a sharp window of expression (8–12 dpp) during the period of exponential fruit growth, coinciding with peak cuticle and wax deposition (**Figure 5B**). Consistent with observed differences in chromosome 1-associated traits of cuticle thickness, intercalation depth, and diameter of lipid droplets, expression of *CsSHN1* was significantly higher in the two pickling cultigens, Gy14 and Vlaspik (Ando et al., 2015), than in CL9930. Peak expression was also somewhat delayed in CL9930, at 12 dpp vs. 8 dpp, corresponding with the relative timing for increase in cuticle thickness, intercalation depth, and diameter of lipid droplets.

The predicted length of *CsSHN1* is 957 bp; transcript data (http://cucurbitgenomics.org/) support a single intron, consistent with other *SHINE* genes (Borisjuk et al., 2014). Comparison of the coding region plus 2 Kb upstream between Gy14 and CL9930 identified a SNP, within exon 2. The Vlaspik and Poinsett 76 sequences also shared the Gy14 sequence. A KASP marker was designed for the SNP at position 16961026 on chromosome 1 (CL9930 v. 3). The allele present (CL9930 vs. Gy14) at this position in the RILs completely co-segregated with phenotype. Marked allele effects were observed for the four fruit epidermal traits (**Figure 5C**).

The SNP at this position (‘C’ in Gy14 vs. ‘G’ in CL9930) results in a predicted amino acid change, from proline in Gy14 to arginine in CL9930, within a highly conserved region of the protein [domain CMV-1 as per Nakano et al. (2006)]. All of the other cucurbits for which there are draft genomes [*Citrullus lanatus*, *Cucumis melo*, *Cucurbita maxima*, *Cucurbita moschata*, *Cucurbita pepo*, *Cucurbita argyrosperma*, *Lagenaria siceraria* (http://cucurbitgenomics.org/)] like Gy14, contain proline in this position (**Supplementary Table 5**). In addition, more than 30 divergent plant species with homologs identified by BLAST, also contain a proline residue at this position (**Supplementary Table 7**). Within cucumber germplasm, however, the CL9930 variant is quite common. Of 140 re-sequenced accessions with ≥10 reads at this position, 44 exhibited the CL9930 allele; another nine are heterozygous at this position (**Supplementary Table 4**).

## Discussion

### Variation in Epidermal Properties of Cucumber Fruit During Development

Cucumber fruit sampled at incremental ages from anthesis through maturity were characterized for developmental changes and natural variation for epidermal traits, including epidermal cell shape, cuticle thickness, cuticular intercalations between epidermal cells, and the number and size of lipid droplets. Fruits from both Gy14 and CL9930 followed a characteristic sigmoidal pattern of growth, consistent with previous studies (Colle et al., 2017). The associated epidermal fruit traits also exhibited this pattern, with the greatest increase occurring at 4–12 days post pollination, coinciding with the period of exponential growth. The period of peak deposition of cuticle and wax during the period of maximal cucumber fruit growth is consistent with other systems where cuticle and wax deposition is developmentally programmed, often ceasing during early fruit development (Hen-Avivi et al., 2014; Martin and Rose, 2014; Lara et al., 2015; Trivedi et al., 2019). Beginning with the commencement of exponential growth and continuing throughout development, Gy14 had consistently larger values for cuticle thickness, cuticular intercalation, and number and size of lipid droplets. By 16 dpp, fruit size and differences in epidermal traits had largely plateaued; therefore, 16–20 dpp became the benchmark age for further epidermal work.

A striking observation was the presence of numerous large lipid droplets, typically 4–10 µm, in the epidermal cells. In plants, lipid droplets are thought to be formed in the endoplasmic reticulum and surrounded by a monolayer of phospholipids and structural membrane proteins (Chapman et al., 2012; Huang, 2018; Shimada et al., 2018). Lipid droplets in plants can vary quite widely in size, ranging from <1 to ∼20 µm, depending on species and organ or tissue; larger ones are more frequently found in oil rich fruit tissues (Goold et al., 2015). Much of what is known about the roles of lipid droplets comes from research involving seeds and leaves, but studies of fruits of avocado, olive, and oil palm suggest that the lipid droplets likely have varying functions for different tissue types (Pyc et al., 2017). Originally, lipid droplets were thought to have functions restricted to lipid storage, but recent findings have suggested that lipid droplets can be involved in more complex processes, such as lipid signaling and disease resistance (Chapman et al., 2012; Pyc et al., 2017). Lipid droplets also can sequester lipid-soluble compounds such as terpenoids that may contribute to protection against fungal or oomycete pathogens (e.g., Shimada et al., 2018; Sadre et al., 2019). Whether the cucumber fruit lipid droplets function in other capacities such as defense remains to be investigated.

### Mapping of, and Relationships Among, Epidermal Fruit Traits

Phenotypic analysis of the Gy14 × CL9930 RIL populations was performed to ascertain genetic factors underlying the variation in epidermal traits. QTL for the six epidermal traits were detected on six of the seven cucumber chromosomes. Given the large LOD profiles and high correlation of traits mapping to chromosome 1, SSR markers designed to cover the peak QTL region on chromosomes 1 were used to screen an expanded Gy14 × CL9930 F_7:8_ RIL population for recombinants in this region. Phenotyping of identified recombinants were narrowed to a region of ∼3 Mb on chromosome 1. Fine mapping of the region of interest on chromosome 1 using KASP^™^ markers narrowed the region to an area of ∼0.5 Mb. The very strong correlation between phenotype traits in the field and greenhouse indicate that, despite greatly differing environments, the measured traits on chromosome 1 are predominantly affected by genotype and developmental stage, rather than environment.

Strong, positive correlations were observed for cuticle thickness, intercalation depth, epidermal cell height, and diameter of lipid droplets along with a strong QTL on chromosome 1. While there was variation among RILS for relative intercalation depth (i.e., not all long cells had deep intercalations) the very strong correlation between epidermal cell height and intercalation depth argues that cell structure may be an important factor influencing cuticular intercalations in cucumber fruit. Diversity in intercalation patterns has been observed in a variety of species as illustrated by a ‘grocery store survey’ of numerous fruit types (Martin and Rose, 2014). The origin of such material remains unclear, possibly due to detachment from the epidermal cuticle and downward movement, or direct deposition to the anticlinal cell wall regions. It is also not clear whether diversity in intercalation patterns result from active regulation or from mechanical constraints of the cell structure (Martin and Rose, 2014). Consistent with a possible role of cell structure, in addition to the major QTL for intercalation depth and epidermal cell height on cucumber chromosome 1, intercalation depth also appears to share a QTL with epidermal cell height and cell width on chromosome 4.

Epidermal cell height and width showed a modest, negative association, and RIL phenotyping displayed a wide range of cell shapes beyond that of the flat and palisade orientations characteristic of CL9930 and Gy14, respectively. This variation is likely due to multiple factors controlling epidermal cell shape, including the *Pe* gene on chromosome 5. *Pe* has been localized to a 0.23 Mb region and exhibits tight, but not unbreakable, linkage to several fruit surface related traits such as dull (*D*), uniform fruit color (*u*) and tuberculate (*Tu*), suggesting a cluster of genes modulating cucumber exocarp characteristics (Yang et al., 2014a, Yang et al., 2014b, Yang et al., 2014c; Chen et al., 2016; Zhang et al., 2017; Yang et al., 2019). The QTL for epidermal cell width identified on chromosome 5 in this study is consistent with the location of *Pe*. Interestingly, the number of lipid droplets was not significantly related to size of lipid droplets suggesting that multiple factors regulate lipid droplet formation. Consistent with this observation, QTL for number of lipid body number and size were present on different chromosomes, 4 and 6 for number, and 1, 2 and 6, for size.

### *CsSHN1* is a Candidate Gene Influencing Cucumber Fruit Surface Properties

Mapping results and SSR and KASP marker assay refined the major QTL on chromosome 1 to a region containing 25 annotated genes. Expression profiles of these genes showing peak transcription coinciding with period of rapid fruit growth and deposition of cuticle, strongly preferential expression in fruit exocarp, and known function of SHINE transcription factors as regulators of cuticle and wax deposition (Yeats and Rose, 2013; Hen-Avivi et al., 2014; Trivedi et al., 2019), collectively implicate *CsSHN1* (Csa1g340430) as the primary candidate gene underlying the chromosome 1 QTL. *SHN* (*SHINE*) or *WIN* (*WAX INHIBITOR*) genes are members of the apetala2/ethylene-responsive element biniding protein (ap2/ere bp) transcription factor family originally named in Arabidopsis for their role in leaf appearance and the regulation of cuticle biosynthesis (Aharoni et al., 2004; Broun et al., 2004). *SHN* genes are primarily expressed in epidermal tissue in locations and periods of rapid growth, allowing for coverage and protection of the developing organ (Hen-Avivi et al., 2014; Trivedi et al., 2019). Expression of *CsSHN1* in cucumber fruit was consistent with this pattern, and mirrors the tissue specific and developmental regulation observed for *SlSHN3* in tomato fruit (Shi et al., 2013). Similar to *SlSHN3, CsSHN1* is nearly exclusively expressed in exocarp of immature fruit relative to other organs, tissues and ages.

Several studies have demonstrated that overexpression of *SHN* homologs increases wax deposition and cuticle thickness by modulating expression of cutin and wax biosynthesis genes, either directly or indirectly; conversely, down-regulation results in reduced cuticle and waxes (e.g., Aharoni et al., 2004; Broun et al., 2004; Kannangara et al., 2007; Shi et al., 2013). Variants for cuticle and wax deposition have primarily identified by mutant screens; however, more recent genomic, transcriptomic and metabolomic approaches have enabled the identification of natural variants (Cohen et al., 2017). While the majority of cuticle and wax variants identified to date include biosynthetic enzymes and lipid transporters [e.g., fatty acid omega hydroxylase (CYP861A, CYP86B1), BAHD acetyltransferase, beta-ketoacyl-CoA synthase, triterpene synthases, GDSL lipase] (Cohen et al., 2017), it has been suggested that regulatory genes are the most likely targets to achieve fine modulation (Petit et al., 2017). Naturally occurring variation for the naked caryopsis phenotype in barley, a trait causing loss of a sticky lipid substance secreted by the epidermis, was found to arise from mutation in a *SHN1* allele in barley (Taketa et al., 2008; Taketa et al., 2012) and genomic studies in apple have suggested that variations in the apple homolog of *SHN1* (*MdSHN3*), influence cuticle formation and russeting disorder in apple fruit (Lashbrooke et al., 2015b).

CsSHN1, like other SHINE and AP2/EREBP proteins includes the highly conserved ERF domain in the amino terminal portion of the protein. SHINE proteins are assigned to Group V of the AP2/EREBP family (Nakano et al., 2006; Borisjuk et al., 2014). Group V includes a single intron and two conserved domains, CMV-1 and CMV-2, toward the middle and C-terminal portion of the protein, respectively. These features also occur in CsSHN1. The substitution of arginine for proline in CL9930 vs. Gy14 occurs within the conserved CMV-1 domain [also referred to as middle motif ‘mm’ (Aharoni et al., 2004)]. Mutation of a valine to aspartic acid mutation in this motif was shown to cause the naked caryopsis phenotype in barley, indicating functional significance of this domain (Taketa et al., 2008; Taketa et al., 2012). It remains to be determined whether the observed mutation in *CsSHN1* influences activity of the CsSHN1 transcription factor. The phenotypic differences observed between Gy14 and CL9930 may reflect protein activity and/or expression levels, as CL9930 also had reduced expression relative to Gy14. We did not observe sequence differences within the promoter (2 kb upstream of the coding region) or intron, suggesting that effects on transcript levels may result from other more distant elements or from relative RNA stability.

Despite conservation of the proline at this position among more than thirty species examined, including both dicots and monocots, the substitution was quite common among cucumber accessions (present in approximately a third of the re-sequenced lines). Cucumber is thought to have been first domesticated in South Asia and then subsequently moved both east toward China and west toward Europe, forming three major clades (Lv et al., 2012; Qi et al., 2013; Wang et al., 2018). Although there are exceptions, the CL9930 allele is predominantly found (70%) in East Asian accessions where it is widely present in cultivated East Asia cucumbers, but not landraces. This may suggest that this gene is under selection in the making of East Asia cucumbers (long, thin skin). The more frequent Gy14 allele is in present in many cultivars, landraces, semi-wild and wild cucumbers. Interestingly, though, it appears that the CL9930 allele also may be present at a relatively low frequency in the wild *C. sativus* var. *hardwickii*, as one of the 12 re-sequenced *hardwickii* accessions possessed this variant. This may reflect possible occurrence prior to domestication. Alternatively, as gene flow between cultivated cucumber and *hardwickii* populations occurs in natural populations (Bisht et al., 2004; Yang et al., 2012), this variation may have originated after domestication.

Several lines of evidence additionally suggest interplay between cuticle deposition and epidermal cell differentiation and development (e.g., Javelle et al., 2010; Nadakuduti et al., 2012; Yeats and Rose, 2013; Hen-Avivi et al., 2014; Fernandez-Moreno et al., 2017). This also has been observed for members of the *SHINE* family. For example, overexpression of Arabidopsis *SHN1* altered epidermal cell structure, including formation of elongated cells and reduced stomatal density and trichome number (Aharoni et al., 2004) and tomato *SlSHN3* influences cell shape, either directly, or indirectly by influencing expression of other cell downstream patterning genes such as *SlMIXTA* (Shi et al., 2013; Lashbrooke et al., 2015a). Down regulation of *SlSHN3* results in reduced cuticle deposition and flattened fruit epidermal cells. The connection between *SHINE*-family member genes and cell shape may also contribute to the observed QTL for cucumber epidermal cell height at this location.

## Conclusions

Cucumber fruit epidermis exhibits dynamic developmental changes during fruit growth including changes in cell size and shape, deposition of cuticle, and appearance of lipid droplets. There is also natural variation for these traits as manifest in differing cucumber market classes, and observed for the Chinese fresh market cucumber, CL9930, relative to the American pickling cucumber, Gy14. Genetic analyses indicated several QTL, including a major QTL on chromosome 1, QTL *ECT1.1*, influencing cuticle thickness and depth of intercalation between epidermal cells, diameter of lipid droplets and epidermal cell height. Additional QTL of lesser impact were present on chromosomes 3, 4, 5 and 6. Fine mapping of the four traits associated with QTL *ECT1.1* narrowed the region to 0.5 Mb. Transcriptomic analysis based on tissue-specific and developmentally-regulated expression of fruit epidermal traits of genes in this region along with and observed allelic effects, identified a primary candidate gene—a homolog of *SHINE1,* which in other systems has been shown to influence both cuticle deposition and epidermal cell shape. The *CsSHN1* sequence in CL9930 includes a single base difference causing an amino acid change (proline to arginine) in the highly conserved CMV-1 domain when compared to that in Gy14. This single base change, which occurred frequently in East Asian cucumber accessions may contribute to natural variation for cucumber epidermal properties. As epidermal properties, including wax deposition, influence both consumer preferences and longevity in the market chain, allelic variation in *CsSHN1* may provide a valuable target for breeders developing varieties to meet desired fruit quality characteristics, such as fruit with shinier appearance (reduced wax) or extended shelf life due to reduced water loss (increased wax).

## Data Availability Statement

The datasets generated for this study can be found in the NCBI Sequence Read Archive (SRA) under project accession number PRJNA558838.

## Authors Contributions

SR-C, RG and CB conceived of the project. SR-C and RG wrote the paper. SR-C performed the developmental analyses, phenotyping of the RILs, QTL mapping, KASP analyses and fine mapping. MC performed the developmental analysis of CsSHN1 expression. BM performed the RNA-Seq experiment, differential gene expression analysis and SNP calling of parental lines. YWa and YWe performed the SSR analysis and identification of recombinants. LG and ZF analyzed the re-sequenced cucumber accessions for alleles of CsSHN1.

## Funding

This research was in part supported by BARD, The United States–Israel Binational Agricultural Research and Development Fund, Research Grant Award No. US-5009-17; the National Institute of Food and Agriculture (NIFA), U.S. Department of Agriculture, Award No. 2015-51181-24285, and by USDA NIFA Hatch project number MICL02349 to RG and MICL02552 to CB.

## Conflict of Interest

The authors declare that the research was conducted in the absence of any commercial or financial relationships that could be construed as a potential conflict of interest.
