# Supplementary material for: QTL and Transcriptomic Analyses Implicate Cuticle Transcription Factor SHINE as a Source of Natural Variation for Epidermal Traits in Cucumber Fruit
Source: Front Plant Sci. 2019 Nov 27;10:1536. doi: 10.3389/fpls.2019.01536 (PMC6890859; doi:10.3389/fpls.2019.01536)
Supplement: Supplementary file 1 [file Image_1.pdf]

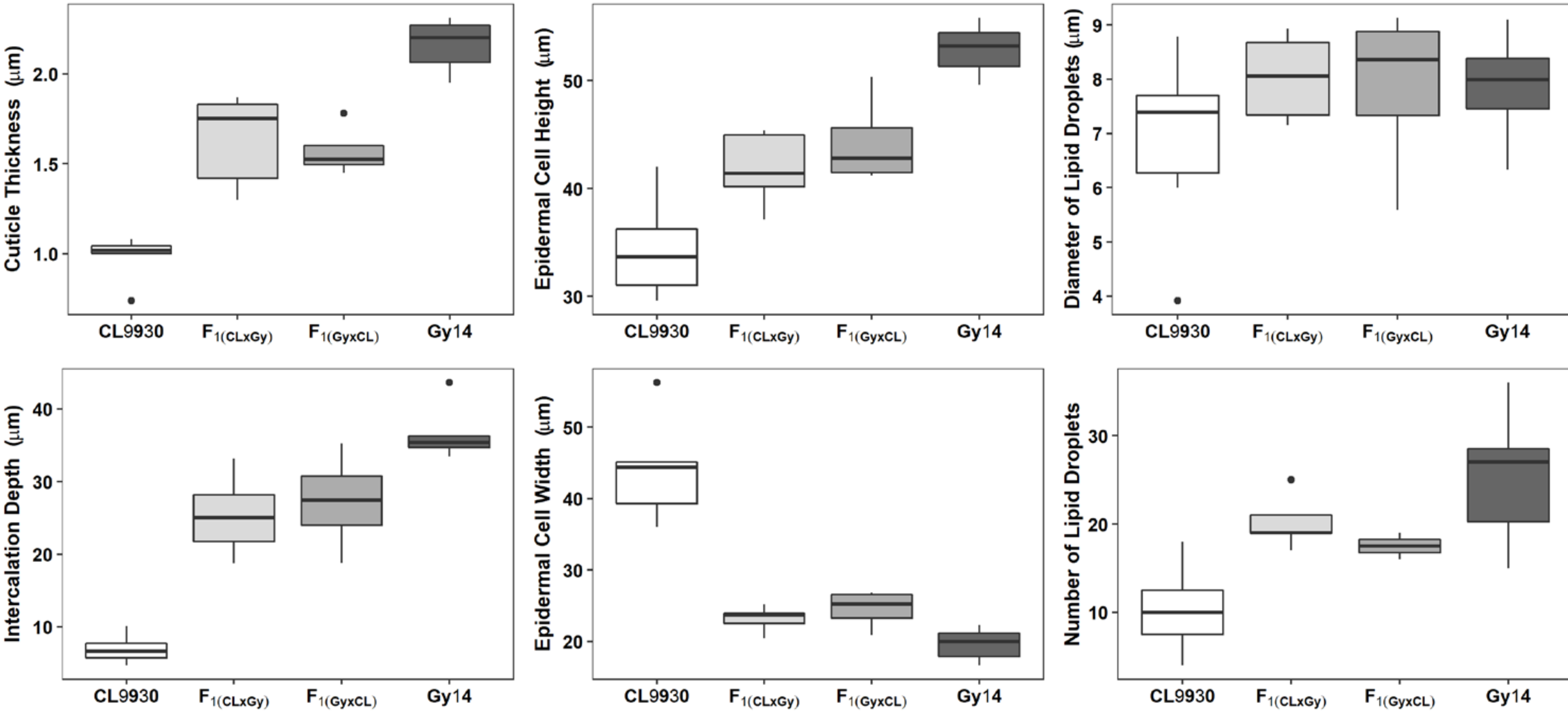

Supplementary Figure 1. Epidermal cell trait phenotypes for parents, Gy14, CL9930 and their reciprocal F_1 hybrids (Gy14 x CL9930) and (CL9930 x Gy14).
