# Supplementary material for: QTL and Transcriptomic Analyses Implicate Cuticle Transcription Factor SHINE as a Source of Natural Variation for Epidermal Traits in Cucumber Fruit
Source: Front Plant Sci. 2019 Nov 27;10:1536. doi: 10.3389/fpls.2019.01536 (PMC6890859; doi:10.3389/fpls.2019.01536)

Supplementary Table 1. Cucumber lines used in the epidermal trait study.

| Line | Type |  | Fruit shape (Length/diameter ratio) at 16-20 dpp | Cuticle thickness at 16-20 dpp^a^ |
| --- | --- | --- | --- | --- |
| Gy14 | American pickling | Inbred breeding line | 2-3 | 2.16 + 0.06 |
| Vlaspik | American pickling | Hybrid variety | 2-3 | 2.29 + 0.05 |
| Poinsett 76 | American slicing | Inbred variety | 3-4 | 2.90 + 0.09 |
| CL9930 | Chinese fresh market | Inbred breeding line | 6-7 | 0.98 + 0.05 |

^a^ Each value is the mean of 3 or 6 biological replicates with 3 technical replicates/biological

replicate + S.E.

Photos of field grown fruit (Summer 2019) harvested at 16-20 days post pollination (dpp).


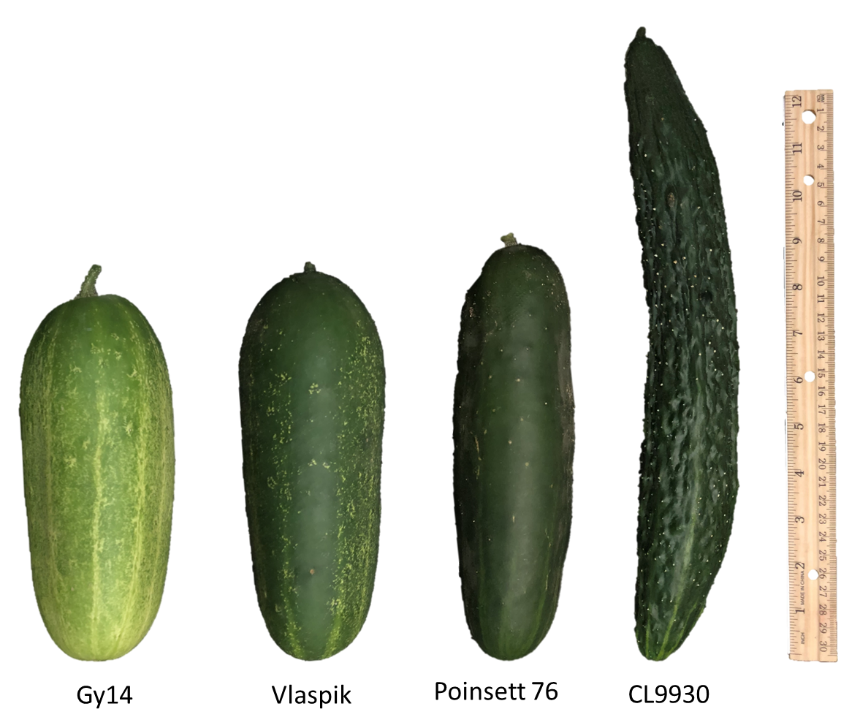

Supplement: Supplementary file 2 [file Table_1.docx]
