# Supplementary material for: QTL and Transcriptomic Analyses Implicate Cuticle Transcription Factor SHINE as a Source of Natural Variation for Epidermal Traits in Cucumber Fruit
Source: Front Plant Sci. 2019 Nov 27;10:1536. doi: 10.3389/fpls.2019.01536 (PMC6890859; doi:10.3389/fpls.2019.01536)
Supplement: Supplementary file 3 [file Table_2.docx]

**Supplementary Table 2.** SSR markers used for screening of Gy14 × 9930 RILs.

| **Markers** | **Chr** | **Gy14V2.0** | **9930_V2.0** | **9930_V3.0** | **Left** | **Right** |
| --- | --- | --- | --- | --- | --- | --- |
| **UW083733** | Chr1 | 18212935 | 14833061 | 18050191 | TTGGATCTGTGAAATTCAGTTTG | TCGTGAAGGGCTCTTTATAGG |
| **UW005624** | Chr1 | 14730848 | 12036630 | 14516668 | GAATGGTGGAAGGGAAGTGA | GGTGATTGGAAGAGTGTGGG |
| **UW053209** | Chr1 | 14879475 | 11888310 | 14783187 | GGGCAAAAGATGACAATCCA | TGTCATCCTGCAATTAAGCG |
| **UW084513** | Chr1 | no hit | 11611700 | 12804673 | TTGAAATGGATTTGTGGCTTA | TGCACTTTTATTTTTATTTATGACAAC |
| **UW084513** | Chr1 | no hit | 11611700 | 12804673 | TTGAAATGGATTTGTGGCTTA | TGCACTTTTATTTTTATTTATGACAAC |
| **UW084438** | Chr4 | 17901764 | 10876828 | 13618421 | TTGGGTATCACATGCCATAAA | ATTGTTTGGCATTCCCACAT |
| **UW084529** | Chr4 | 10307908 | 9795426 | 9958625 | TCCTCACAACTAAATACACATAACAA | CTGATTAATGAATATGAAGTTGACACA |
| **SSR06253** | Chr4 | 9004636 | 8589928 | 8768561 | TGTGGGTACTCCTCAAACCA | TGATATCCATATCTTTGAAATGTCTTT |
| **UW058481** | Chr4 | 6530090 | 6302670 | 6427570 | GGATTGAGGATTTTGCTGGA | CGGAGACAAAATGGACTGCT |
