# Supplementary material for: QTL and Transcriptomic Analyses Implicate Cuticle Transcription Factor SHINE as a Source of Natural Variation for Epidermal Traits in Cucumber Fruit
Source: Front Plant Sci. 2019 Nov 27;10:1536. doi: 10.3389/fpls.2019.01536 (PMC6890859; doi:10.3389/fpls.2019.01536)
Supplement: Supplementary file 7 [file Table_6.docx]

**Supplementary Table 6.** Correlation and corresponding p-values for epidermal traits of cucumber fruit grown under field (Summer 2018) and greenhouse (Spring 2019) conditions

| **Spring 2019 - Greenhouse** | **Summer 2018 – Field** | | | | |
| --- | --- | --- | --- | --- | --- |
|  |  | **CT**  Cuticle thickness | **ID**  Intercalation depth | **DLD**  Diameter of lipid droplets | **ECH**  Epidermal cell height |
|  | **CT** | 0.985^a^  1.62E-14^b^ |  |  |  |
|  | **ID** |  | 0.956 ^a^  1.75E-10 ^b^ |  |  |
|  | **DLD** |  |  | 0.839 ^a^  7.23E-06 ^b^ |  |
|  | **ECH** |  |  |  | 0.863 ^a^  2.00E-06 ^b^ |

^a^Pearson correlation coefficient. N=19; 17 RILs and parental Gy14 and CL9930. All values were the mean of three biological replicates with three technical replicates.

^b^p-value of correlation. Pearson correlation coefficients and their corresponding p-values were calculated in R (version 3.6.0;

<https://www.r-project.org>) using the ‘Hmisc’ package (<https://cran.r-project.org/web/packages/Hmisc/>).
