# Supplementary material for: QTL and Transcriptomic Analyses Implicate Cuticle Transcription Factor SHINE as a Source of Natural Variation for Epidermal Traits in Cucumber Fruit
Source: Front Plant Sci. 2019 Nov 27;10:1536. doi: 10.3389/fpls.2019.01536 (PMC6890859; doi:10.3389/fpls.2019.01536)
Supplement: Supplementary file 8 [file Table_7.docx]

| **Supplementary Table 7.** Alignment of the conserved CMV-1 domain (as per Nakano et al., 2006) of the SHINE1 protein across species. | |
| --- | --- |
|  | * |
| *Cucumis sativus – Gy14* | GLSEILQAKLKKC-CRTPS**P**SMTCLRLDTENSNIGVWQKRAG |
| *Cucumis sativus – CL9930* | GLSEILQAKLKKC-CRTPS**R**SMTCLRLDTENSNIGVWQKRAG |
| *Cucumis melo* | GLSEILQAKLKKC-CRTPS**P**SMTCLRLDTENSNIGVWQKRAG |
| *Cucurbita moschata* | GLSEILQAKLKKC-CRTPS**P**SMTCLRLDTENSNIGVWQKRAG |
| *Cucurbita pepo* | GLSEILQAKLKKC-CRTPS**P**SMTCLRLDTENSNIGVWQKRAG |
| *Monmordica charantia* | GLSQMLQAKLKKWRCRAPS**P**SMTCLRLDTENSNIGVWHKCAG |
| *Quercus suber* | GLSEILHAKLRKC-SKAPS**P**SMTCLRLDTENSHIGVWQKRAG |
| *Citrus sinesis* | ELSQLLHAKLRKC-SKTPS**P**SMTCLRLDTENSHIGVWQKRAG |
| *Juglans regia* | DLSEILHAKLRKC-SKAPS**P**SMTCLRLDTVSSHIGVWQKRAG |
| *Ricinus communis* | GLSEILHAKLRKC-SKTPS**P**SMTCLRLDTENSHIGVWQKRAG |
| *Malus domestica* | GLSEILHAKLRKC-SKIPS**P**SMTCLRLDNESSHIGVWQKRAG |
| *Manihot esculenta* | GLSEILHAKLRKC-SKTPS**P**SMTCLRLDTENSHIGVWQKRAG |
| *Havea brasiliensis* | GLSAILHAKLRKC-GKAPS**P**SMTCLRLDTENSHIGVWQKRAG |
| *Gossypium raimondii* | ELSELLHAKLRKC-SKAPS**P**SMTCLRLDTENSHIGVWQKRAG |
| *Lupinus angustifolius* | DLEEILHAKLRKC-GKVPS**P**SMTCLRLDTENSHIGVWQKRAG |
| *Phaseolus vulgaris* | DLEEILHAKLRKC-GKVPS**P**SMTCLRLDTENSHIGVWQKRAG |
| *Nicotiana tabacum* | ALSEILYAKLRKC-SKVPS**P**SLTCLRLDIENSHIGVWQKRAG |
| *Glycine max* | DLEEILHAKLRKC-SKVPS**P**SMTCLRLDTENSHIGVWQKRAG |
| *Solanum tuberosum* | ALSEILHAKLRKC-SKVPS**P**SLTCLRLDIESSHIGVWQKRAG |
| *Coffea arabica* | DLSEILHAKLRKC-SKAPS**P**SLTCLRLDIENSNIGVWQKRAG |
| *Zea mays* | SLSQILSAKLRRC-CKTPS**P**SLTCLRLDPEKSHIGVWQKRAG |
| *Lycopersicum esculentum* | SLSSILSAKLRKC-CKSPS**P**SLTCLRLDTASSHIGVWQKRAG |
| *Arabidopsis thaliana* | SLSSILSAKLRKC-CKSPS**P**SLTCLRLDTASSHIGVWQKRAG |
| *Oryza sativa* | NLSQILSAKLRKC-CKAPS**P**SLTCLRLDPEKSHIGVWQKRAG |
| *Zea mays* | SLSQILSAKLRRC-CKTPS**P**SLTCLRLDPEKSHIGVWQKRAG |
